# Supplementary material for: Anti-microRNA-21 Therapy on Top of ACE Inhibition Delays Renal Failure in Alport Syndrome Mouse Models
Source: Cells. 2022 Feb 9;11(4):594. doi: 10.3390/cells11040594 (PMC8869926; doi:10.3390/cells11040594)
Supplement: Supplementary file 1 [file cells-11-00594-s001.zip › cells-1518663-supplementary.pdf]

Supplementary Materials:

Table S1: Survival in 129/SvJ *Col4a3*$^{-/-}$ mice in weeks

Group	No of animals	Mean	±	SEM	P
Veh	8	10.0	±	0.4	
Anti-miR-21	8	11.4	±	0.5	
ACEi	8	15.3	±	1.4	* #
ACEi+Anti-miR-21	8	21.6	±	2.0	* # §

* P<0.05 vs. Veh, # P<0.05 vs. Anti-miR-21, § P<0.05 vs. ACEi

Table S2: Relative Proteinuria (fold vs. Veh) in 129/SvJ *Col4a3*$^{-/-}$ mice

		High molecular weight proteinuria					Albuminuria				
Group	Age of animals (weeks)	No of animals	Mean	±	SEM	P	No of animals	Mean	±	SEM	P
WT	9.5	7	0.04	±	0.02		7	0.02	±	0.02	
Veh	4.5	3	0.37	±	0.17		4	0.97	±	0.22	
Veh	6.0	4	1.32	±	0.50		4	1.24	±	0.28	
Veh	7.5	4	1.59	±	0.33		4	1.11	±	0.23	
Veh	9.5	4	0.90	±	0.25	*	4	0.92	±	0.14	*
Anti-miR-21	4.5	4	0.88	±	0.15		4	0.68	±	0.15	
Anti-miR-21	6.0	3	0.56	±	0.05		4	0.74	±	0.15	
Anti-miR-21	7.5	3	0.64	±	0.12	§	4	0.94	±	0.15	
Anti-miR-21	9.5	4	0.47	±	0.15		4	1.00	±	0.22	*
ACEi	4.5	3	0.04	±	0.01		4	0.16	±	0.08	
ACEi	6.0	3	0.04	±	0.02	#	3	0.60	±	0.41	
ACEi	7.5	3	0.15	±	0.03	§	4	0.71	±	0.26	
ACEi	9.5	3	0.21	±	0.17		4	0.73	±	0.41	
ACEi+Anti-miR-21	4.5	4	0.11	±	0.07		3	0.38	±	0.24	
ACEi+Anti-miR-21	6.0	4	0.11	±	0.05	#	4	0.45	±	0.15	
ACEi+Anti-miR-21	7.5	4	0.04	±	0.01	§	4	0.14	±	0.05	§
ACEi+Anti-miR-21	9.5	4	0.16	±	0.03		4	0.52	±	0.15	

* P<0.05 vs. WT 9.5w, # P<0.05 vs. Veh 6.0w, § P<0.05 vs. Veh 7.5w

Table S3: BUN in 129/SvJ *Col4a3*$^{-/-}$ mice in mg/dl

Group	Age of animals (weeks)	No of animals	Mean	±	SEM	P
Veh	7.5	8	73.4	±	9.2	
Veh	9.5	7	460.1	±	60.8	
Anti-miR-21	7.5	8	72.7	±	9.5	
Anti-miR-21	9.5	5	304.0	±	106.0	*
ACEi	7.5	4	42.5	±	3.4	
ACEi	9.5	2	45.6	±	5.7	* #
ACEi+Anti-miR-21	7.5	4	42.0	±	5.4	
ACEi+Anti-miR-21	9.5	3	60.6	±	3.6	* #

* P<0.05 vs. Veh 9.5w, # P<0.05 vs. Anti-miR-21 9.5w

Table S4: Kidney Pathology in 129/SvJ *Col4a3*$^{-/-}$ mice

		Laminin Staining										Fibronectin Staining									
		Glomerular score					Tubulointerstitial score					Glomerular score					Tubulointerstitial score				
Group	Age of animals (weeks)	No of values	Mean	±	SEM	P	No of values	Mean	±	SEM	P	No of values	Mean	±	SEM	P	No of values	Mean	±	SEM	P
WT		84	0.5	±	0.1		75	0.6	±	0.1		69	0.3	±	0.1		69	0.1	±	0.0	
Veh	7.5	75	1.3	±	0.1		69	1.08	±	0.1		63	1.2	±	0.1		63	0.9	±	0.1	
Veh	9.5	68	1.9	±	0.1		60	2.05	±	0.1		72	2.2	±	0.1		72	1.5	±	0.1	
Anti-miR-21	7.5	84	1.8	±	0.1	*	87	1.4	±	0.1	*	73	1.6	±	0.1	*	75	0.7	±	0.1	
Anti-miR-21	9.5	93	2.3	±	0.1	#	90	2.1	±	0.1		72	1.3	±	0.1	#	72	1.0	±	0.1	#
ACEi	7.5	117	1.0	±	0.0	* §	99	0.9	±	0.0	§	78	0.5	±	0.1	* §	78	0.5	±	0.1	*
ACEi	9.5	93	1.1	±	0.0	# $	87	1.2	±	0.1	# $	44	0.3	±	0.1	# $	45	0.4	±	0.1	# $
ACEi+Anti-miR-21	7.5	90	1.2	±	0.1	§ °	69	1.1	±	0.1	§	69	0.3	±	0.1	* §	69	0.3	±	0.1	* §
ACEi+Anti-miR-21	9.5	99	1.1	±	0.0	# $	72	1.2	±	0.1	# $	72	0.5	±	0.1	# $	72	0.5	±	0.1	# $

* P<0.05 vs. Veh 7.5w, # P<0.05 vs. Veh 9.5w, § P<0.05 vs. Anti-miR-21 7.5w, $ P<0.05 vs. Anti-miR-21 9.5w, ° P<0.05 vs. ACEi 7.5w

Table S5: Survival in males F1-*Col4a3*$^{-/-}$ in weeks

Group	No of animals	Mean	±	SEM	P
Veh	12	16.6	±	0.2	
Anti-miR-21	12	22.1	±	0.5	* §
ACEi	12	18.9	±	0.5	* #
ACEi+Anti-miR-21	12	24.0	±	0.9	* §

* P<0.05 vs. Veh, # P<0.05 vs. Anti-miR-21, § P<0.05 vs. ACEi

Table S6: BUN in males F1-*Col4a3*$^{-/-}$ mice in mg/dl

Group	BUN mg/dL (Mean±SEM)							
	5 weeks	7 weeks	9 weeks	11 weeks	13 weeks	15 weeks	17 weeks	19 weeks
Wildtype	26.6±0.6 n=12	23.2±0.9 n=12	24.4±0.5 n=12	24.2±0.5 n=12	23.0±0.5 n=12	24.1±0.6 n=12	NC	NC
Veh	25.9±0.5 n=12	25.9±1.0 n=12	35.9±2.6 n=12	53.9±4.2 n=12	72.2±5.2 n=12	135.9±13.8 n=11	NC	NC
p-value	NS	*	*	*	*	*		
Anti-miR-21	25.3±0.3 n=12	24.2±0.5 n=12	27.6±1.0 n=12	30.5±1.1 n=12	36.3±1.9 n=12	49.1±3.2 n=12	78.4±9.1 n=12	132.3±17.0 n=12
p-value	NS	NS	#	#	#	#		
ACEi+Anti-miR-21	26.2±0.5 n=12	25.1±0.6 n=12	27.1±0.9 n=12	29.3±1.4 n=12	34.6±3.0 n=12	46.3±5.6 n=12	71.0±8.5 n=11	91.0±7.4 n=11
p-value	NS	NS	&	&	&	&		
ACEi	27.6±0.7 n=12	25.2±0.7 n=12	29.8±1.6 n=12	38.7±3.2 n=12	52.3±4.6 n=12	87.8±11.5 n=12	159.9±29.9 n=10	203.4±30.8 n=5

NC Blood not collected

NS=non significant, * P<0.05 vs. WT, # p<0.05 vs. Veh,
&p<0.05 vs Veh,

Table S7: BUN in males F1-*Col4a3*$^{-/-}$ mice in mg/dl

Group	BUN mg/dL (Mean±SEM)			
	6 weeks	9 weeks	12 weeks	15 weeks
Wildtype	28.1±0.8 n=5	24.8±0.5 n=5	25.3±1.3 n=5	25.5±0.7 n=5
Veh	26.0±0.5 n=7	29.6±1.3 n=7	58.1±7.9 n=7	121.2±15.6 n=7
p-value	NS	NS	*	*
Anti-miR-21	25.8±0.8 n=7	22.8±1.3 n=7	25.7±0.8 n=7	33.1±2.5 n=7
p-value	NS	&	&	*&
ACEi	28.0±0.6 n=6	27.5±0.7 n=6	36.1±3.4 n=6	60.9±7.0 n=6
p-value	NS	NS	*	*&

NS=non significant, * P<0.05 vs.WT, & p<0.05 vs Veh,

Table S8: Relative kidney miR-21 levels normalized to miR-191 in males F1-*Col4a3*$^{-/-}$ mice

Group	No of animals	Mean	±	SEM	P
WT	6	1.01	±	0.03	
Veh	6	7.73	±	0.28	*
Anti-miR-21	6	1.38	±	0.04	*#
ACEi	6	3.83	±	0.26	*#§

* P<0.05 vs. WT, # P<0.05 vs. Veh, § P<0.05 vs. anti-miR-21

Table S9: Kidney pathology scores from males F1-*Col4a3*$^{-/-}$ mice

		Glomerular pathology				Tubular pathology				Interstitial inflammation				Interstitial fibrosis			
Group	No of values	Mean	±	SEM	P	Mean	±	SEM	P	Mean	±	SEM	P	Mean	±	SEM	P
WT	5	0	±	0		0	±	0		0	±	0		0	±	0	
Veh	8	3.9	±	0.1	*	3.8	±	0.1	*	3.5	±	0.2	*	3.3	±	0.1	*
Anti-miR-21	8	2.8	±	0.1	*#	2.3	±	0.1	*#	2.0	±	0.2	*#	2.00	±	0.1	*#
ACEi	6	3.8	±	0.2	*§	3	±	0.1	*#§	2.5	±	0.2	*#	2.5	±	0.2	*#§

* p<0.05 vs. WT, # p<0.05 vs Veh, § p<0.05 vs. Anti-miR-21

		αSMA positive nuclei (%)				Trichrome (surface area mm^2)			
Group	No of values	Mean	±	SEM	P	Mean	±	SEM	P
WT	5	6.4	±	0.7		0.5	±	0.1	
Veh	8	28.5	±	2.6	*	3.4	±	0.4	*
Anti-miR-21	8	8.6	±	0.8	#	1.1	±	0.2	*#
ACEi	6	18.3	±	1.8	*§	1.4	±	0.2	*#

* p<0.05 vs. WT, # p<0.05 vs Veh, § p<0.05 vs. Anti-miR-21

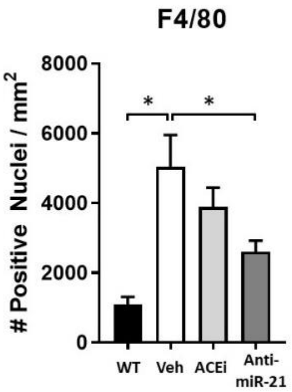

Figure S1. Analysis of F4/80+ nuclei from WT, Veh, ACEi and Anti-miR-21 kidney sections. The number of F4/80 positive nuclei per square mm were quantitated in all animals. *P<0.05 as noted.
